# Estimating Drift Parameters in a Sub-Fractional Vasicek-Type Process

**DOI:** 10.3390/e24050594

**Published:** 2022-04-24

**Authors:** Anas D. Khalaf, Tareq Saeed, Reman Abu-Shanab, Waleed Almutiry, Mahmoud Abouagwa

**Affiliations:** 1 General Directorate of Education in Saladin, Ministry of Education, Tikrit 34001, Iraq; anasdheyab@hust.edu.cn; 2 Nonlinear Analysis and Applied Mathematics (NAAM)-Research Group, Department of Mathematics, Faculty of Science, King Abdulaziz University, P.O. Box 80203, Jeddah 21589, Saudi Arabia; tsalmalki@kau.edu.sa; 3 Mathematics Department, College of Science, University of Bahrain, Sakhir P.O. Box 32038, Bahrain; raboshanab@uob.edu.bh; 4 Department of Mathematics, College of Science and Arts in Ar Rass, Qassim University, Buryadah 52571, Saudi Arabia; wkmtierie@qu.edu.sa; 5 Department of Mathematical Statistics, Faculty of Graduate Studies for Statistical Research, Cairo University, Giza 12613, Egypt

**Keywords:** parameter estimation, Vasicek-type model, strong consistency, central limit theorem, Berry–Esseen, 65Mxx, 34A08, 35A08, 35R13

## Abstract

This study deals with drift parameters estimation problems in the sub-fractional Vasicek process given by $dx_{t}=\theta \left(\mu -x_{t}\right)dt+dS_{t}^{H}$, with θ>0, μ∈R being unknown and t≥0; here, $S^{H}$ represents a sub-fractional Brownian motion (sfBm). We introduce new estimators $\hat{\theta }$ for θ and $\hat{\mu }$ for μ based on discrete time observations and use techniques from Nordin–Peccati analysis. For the proposed estimators $\hat{\theta }$ and $\hat{\mu }$, strong consistency and the asymptotic normality were established by employing the properties of $S^{H}$. Moreover, we provide numerical simulations for sfBm and related Vasicek-type process with different values of the Hurst index *H*.

## 1. Introduction

Modeling a diffusion mechanism that tracks the time evolution of complex phenomena, described by stochastic differential equations (SDEs), is often convenient. Parameters in SDEs are crucial for considering the classification of dynamic phenomena. In most cases, these variables are not precisely defined, although a sample of data is available to specific dynamic occurrences. Generally, more practitioners are interested in using the observation data to obtain accurate estimates of the parameters. In realistic cases, the available data are sampled over a certain time interval by discrete time series data; however, SDEs are almost surely continuous processes. Moreover, no measuring device can consistently track a process trajectory since the process path is too troublesome. Furthermore, El Machkouri et al. [1] and Es-Sebaiy and Viens [2] have shown that many discretization algorithms used to move from continuous trajectory to discrete observation based estimators are inefficient.Therefore, we always prefer to deal with the discreet observed processes directly. All in all, the estimation of parameters for discretely observed processes is not trivial, and it has produced a great deal of research effort over the last few decades.

On the other hand, in 1977, Vasicek introduced a stochastic process, and it was named after him [3]. Since then, the Vasicek process has been employed widely in mathematical finance. Hao et al. [4] utilized it to model the pricing of credit default swaps. In order to explain the dynamics of the short rate in the context of stock warrant pricing, Xiao et al. [5] presented a Vasicek-type process. For more explanation of this work, see also [6,7,8] and the references therein.

The stochastic model of Vasicek-type can be given by the following differential equation.

$$\begin{array}{c} dx_{t}=\theta \;\left(\mu -x_{t}\right)\;dt+dB_{t}^{H};\;\;t\geq 0,\;\;x_{0}=0, \end{array}$$

Here, $B^{H}$ stands for the fractional-Brownian motion (fBm), and from a purely financial standpoint, θ and μ can be taken to mean the following: Parameter μ represents the interest rate’s long-term-average, while parameter θ can be stand for the speed of recovery that $x_{t}$ comes back to μ from the long-term average’s deviation value.

Recently, many scholars have devoted themselves to investigate the problem of the parameter estimation of SDE. Here are some examples of works in this field:

- Ref. [9] considers the drift parameter estimation of the sub-fractional Ornstein–Uhlenbeck process based on continuous observation.
- Parameters estimation for fractional Vasicek process in the ergodic case with continuous time observation has been considered by [10,11,12].
- Es-Sebaiy and Es-Sebaiy [13] and Alazemi et al. [14] explored the problem of parameter estimation in a Vasicek model in the non-ergodic scenario.

Inspired by works mentioned above, in this article, we aim to present and analyse the parameters’ estimation of a sub-fractional Vasicek-type process given via the unique solution for the following SDE:

(1) $$\begin{array}{c} dx_{t}=\theta \;\left(\mu -x_{t}\right)\;dt+dS_{t}^{H};\;\;t\geq 0,\;\;x_{0}=0, \end{array}$$

where $S^{H}$ is a sfBm, which represents an extension of the celebrated fBm. Similarly, process *x* can be defined by the following:

(2) $$\begin{array}{c} x_{t}=\mu \left(1-e^{-\theta t}\right)+e^{-\theta t}\int_{0}^{t}e^{\theta v}dS_{v}^{H};\;\;t,v\geq 0, \end{array}$$

where parameters 0<θ and μ are assumed to be unknown and real.

The drift parameters estimation for Vasicek processes is an essential problem that is both theoretically challenging and well-motivated by practical needs. Our practical reason for looking into this estimation challenge in the context of finance is to develop tools to help scientist understand methods for modeling volatility. In fact, any Vasicek process can be used as a stochastic volatility process.

The following is a breakdown of the paper’s structure. Section 2 discusses some aspects in the calculus of Malliavin in terms of sfBm. In Section 3 and Section 4, the strong consistency and asymptotic behavior of the estimators given to estimate the parameters θ and μ are investigated. Section 5 investigates sample route simulations for the sfBm and the process of sub-fractional Vasicek. Finally, we present proofs for various auxiliary results in the Appendix A.

## 2. Preliminaries

In this section, we recall some sfBm concepts and facts, as well as Wiener space analysis. We begin with a definition of sfBm.

The sub-fractional Brownian motion $S^{H}={\left(S_{t}^{H}\right)}_{t\geq 0}$ is a Gaussian process with zero mean, $S_{0}^{H}=0$, and the covariance is given by the following function [15,16]:

$$r\left(t,v\right):=\;E\left[S_{t}^{H}S_{v}^{H}\right]=v^{2H}+t^{2H}-\frac{1}{2}[{\left(v\;+t\right)}^{2H}{\;+\;|v-t|}^{2H}]$$

for all t,v≥0. Note that, when $H=\frac{1}{2},S^{H}$ corresponds to the well known Brownian motion *B*. Sub-fractional Brownian motion has properties that are similar to those of fractional Brownian motion, such as the following: long–range dependence, Self–similarity, Hölder pathes, and it satisfies [17,18,19,20,21,22,23].

(3) $$\begin{array}{c} E\;\left[S_{t}^{H}-S_{v}^{H}\right]\leq \left[2\;-\;2^{2H-1}\right]{\left|v\;-\;t\right|}^{2H};\;v,t\geq 0, \end{array}$$

It is worth mentioning that according to Kolmogrov’s continuity criterion and Equation (3), sfBm has Hölder continuous pathes of order H−ϵ, for all ϵ∈(0,H).

Now, we let (Ω,ℱ,P) be a Wiener space with the process of Wiener *W*, for any $g,h\in L^{2}\left(R_{+}\right):=ℋ$, we have $E\left[W\left(g\right)W\left(h\right)\right]=<g,h{>}_{ℋ}$ such that that $W\left(g\right):=\int_{R_{+}}g\left(s\right)dW_{s}$.

Define the *q*th Hermite polynomial by $H_{q+1}:=xH_{q}\left(x\right)-H_{q}^{\prime }\left(x\right)$, q≥1, the linear isometry between $H_{q}$ and the symmetric tenser product ${ℋ}^{⊙q}$ can be represented by $I_{q}\left(g^{⊗q}\right):=q!H_{q}\left(W\left(g\right)\right)$ where we have ${∥.∥}_{{ℋ}^{⊙q}}=\sqrt{q!}{∥.∥}_{{ℋ}^{⊗q}}$. *The multiple Wiener integral* of $f_{q}$ with respect to *W* is given by $I_{q}\left(f_{q}\right)$ where $f_{q}\in {ℋ}^{⊙q}$. For the sake of brevity, we will not go over all of the tractable aspects of the multiple Wiener integral; instead, interested readers should consult monograph [24] for a thorough exposition.

Now, we will introduce some basic principles that will be useful throughout this paper.

**First**, *The property of isometry and the product formula*. $\forall g,h\in {ℋ}^{⊙q}$, we have the following.

$$E\left[I_{q}\left(g\right)I_{q}\left(h\right)\right]=q!<g,h{>}_{{ℋ}^{⊗q}}$$

In particular, if *q* equal es one, then the following is the case.

$$\forall g,h\in ℋ,\;I_{1}\left(g\right)I_{1}\left(h\right)=\frac{1}{2}I_{2}\left(g⊗h+h⊗g\right)+<g,h{>}_{ℋ}.$$

**Second**, *A Hypercontractivity property*. ∀q≥1, 2≤p<∞, as well as $g\in {ℋ}^{⊙q}$, then the following is the case:

$$E[|I_{q}{\left(g\right)|}^{p}{]}^{\frac{1}{p}}\leq {\left(p-1\right)}^{\frac{q}{2}}E[|I_{q}\left(g\right){{|}^{2}]}^{\frac{1}{2}}$$

and for q≥1, the following is satisfied:

$${E\;[|F|}^{p}{]}^{\frac{1}{p}}\leq c_{p,q}{E[|\;F\;|}^{2}{]}^{\frac{1}{2}},\;\forall p\geq 2,$$

with $F\in {⊗}_{l=1}^{q}{ℋ}_{l}$; $c_{p,q}$ represents a positive constant.

**Third**, *the distances between random variables*. The total variation distance between the laws of *x* and *y* can be represented as follows:

$$d_{TV}\left(x,y\right)=\;\underset{A\in B(R)}{\sup}\left|P\;\left[x\in A\right]\;-\;P\;\left[y\in A\right]\right|$$

where *x* and *y* are two real-valued random variables and the supremum is taken across all Borel sets. Moreover, if *x* and *y* are two real-valued variables, then the Wasserstein distance between the laws of *x* and *y* is defined as follows.

$$d_{W}\left(x,y\right)=\;\underset{f\in Lip(1)}{\sup}\left|E\;\left[f\left(x\right)\right]-E\;\left[f\left(y\right)\right]\right|,$$

Here, Lip(1) denotes that all functions of Lipschitz with the constant of Lipschitz are less than or equal to one.

## 3. Strong Consistency of Estimators

This section’s main goal is to address the parameter estimation of the process $x={\left(x_{t}\right)}_{t\geq 0}$ defined via (2). In other words, by using Wiener space analysis, we aim to demonstrate the estimators’ strong consistency and asymptotic behavior.

Define $\hat{\mu }:=\frac{1}{n}\sum_{i=1}^{n}x_{i}$. Let 0<H<1, $\lambda_{H}\left(\cdot \right):=4H\Gamma \left(2H\right){\left(\cdot \right)}^{-2H}$, and the following:

(4) $$\begin{array}{c} V:=\frac{1}{\sqrt{n}}\left(\sum_{i=1}^{n}{x_{i}}^{2}-{\left(\sum_{i=1}^{n}x_{i}\right)}^{2}\right), \end{array}$$

then $\hat{\theta }:=\lambda_{H}^{-1}\left(V\right)$, where $\lambda_{H}^{-1}\left(\cdot \right)$ is the inverse function of $\lambda_{H}\left(\cdot \right)$. The process *x* in Equation (2) can be expressed as follows:

(5) $$\begin{array}{c} x_{t}=\mu +\xi_{t}+\gamma_{t}, \end{array}$$

where the following is the case.

(6) $$\begin{array}{c} \xi_{t}:=e^{-\theta t}\int_{-\infty }^{t}e^{\theta s}dS_{s}^{H},\;\gamma_{t}:=-e^{-\theta t}\left(\mu +\xi_{0}\right). \end{array}$$

The process ξ represents a Gaussian noise with co-variance $r_{\xi }\left(k\right):=E\left(\xi_{k}\xi_{0}\right)$, for every k∈Z, where $r_{\xi }\left(0\right):=E{\left(\xi_{0}\right)}^{2}>0$.

Hence, applying (5) and properties of the sfBm (see section 3 in [16]), one obtains, as n→∞

$$\begin{array}{c} \hat{\mu }\;=\;\frac{1}{n}\sum_{i=1}^{n}\;x_{i}\to E\;\left(\mu +\xi_{0}\right)=\mu . \end{array}$$

In what follows, let us consider the expression of (4); it follows from the last equation and Lemma A2 below in the Appendix A that as n→∞, the following is the case.

$$\begin{array}{cc} \frac{1}{n}\sum_{i=1}^{n}{x_{i}}^{2}=\frac{1}{n}\sum_{i=1}^{n}{\left(\mu +\xi_{i}+\gamma_{i}\right)}^{2}\to E{\left(\mu +\xi_{0}\right)}^{2}= & \mu^{2}+r_{\xi }\left(0\right)\\ = & \mu^{2}+\frac{4H\Gamma (2H)}{\theta^{2H}},a.s. \end{array}$$

Now, using definition of $\hat{\theta }$, we obtain the following.

$$\begin{array}{cc} \hat{\theta } & ={\left[\frac{1}{4H\Gamma (2H)}\left(\frac{1}{\sqrt{n}}\sum_{i=1}^{n}x_{i}^{2}-{\left(\frac{1}{n}\sum_{i=1}^{n}x_{i}\right)}^{2}\right)\right]}^{\frac{-1}{2H}}\\ \; & \to {\left[\frac{1}{4H\Gamma (2H)}\left(\mu^{2}+4H\Gamma \left(2H\right)\theta^{-(2H)}-\mu^{2}\right)\right]}^{\frac{-1}{2H}}\\ \; & =\theta . \end{array}$$

By the above discussion, we obtain $\hat{\theta }$ and $\hat{\mu }$, which are strongly consistent; this is the next result’s message.

**Theorem** **1.** *Let 0<H<1 and the process x defined in (1) and (2) be the case; we then have the following:*

(7) $$\begin{array}{c} \left(\hat{\theta },\hat{\mu }\right)\;\;\to \;\;\left(\theta ,\mu \right) \end{array}$$

*a.s. as n converges to +∞.*

## 4. Asymptotic Distribution of $\hat{\theta }$, $\hat{\mu }$

The asymptotic behavior of estimators $\hat{\theta }$ and $\hat{\mu }$ is investigated in this section. We assume that the process *x* in Equation (1) is in the second chaos mode and employ techniques from the calculus of Malliavin.

Now, using the Wiener integral and Hermite polynomial properties, we introduce the following function:

$$\begin{array}{c} \eta_{i}\left(\cdot \right):=e^{\left(i-\cdot \right)}1_{\left(-\infty ,i\right]}\left(\cdot \right), \end{array}$$

hence, ξ in (6) can be given by the following.

$$\begin{array}{c} \xi_{i}=I_{1}\left(\eta_{i}\right). \end{array}$$

Let $R:=\frac{1}{n}\sum_{i=1}^{n}\eta_{i}^{⊗2}$, then $v:=E\left[I_{2}{\left(\sqrt{n}R\right)}^{2}\right]=\frac{2}{n}\sum_{i,j=1}^{n}r_{\xi }{\left(j-i\right)}^{2}$, and we denote the following.

(8) $$\begin{array}{c} F:=I_{2}\left(\frac{\sqrt{n}}{\sqrt{v}}R\right). \end{array}$$

Applying the product formula as well as the process *x* dissection in (5), we obtain the following:

$$\begin{array}{cc} V= & \frac{1}{\sqrt{n}}\sum_{i=1}^{n}x_{i}^{2}-{\left(\frac{1}{n}\sum_{i=1}^{n}x_{i}\right)}^{2}\\ = & \frac{1}{\sqrt{n}}\sum_{i=1}^{n}{\left(\mu +\xi_{i}+\gamma_{i}\right)}^{2}-\frac{1}{n^{2}}\sum_{i,j=1}^{n}{\left(\mu +\xi_{i}+\gamma_{i}\right)}^{2}\\ = & \frac{1}{\sqrt{n}}\sum_{i=1}^{n}\xi_{i}^{2}+L, \end{array}$$

where the following is the case.

(9) $$\begin{array}{c} L:=\frac{2}{\sqrt{n}}\sum_{i=1}^{n}\xi_{i}\gamma_{i}+\frac{1}{\sqrt{n}}\sum_{i=1}^{n}\gamma_{i}^{2}-\frac{1}{n^{2}}\sum_{i,j=1}^{n}\xi_{i}\xi_{j}-\frac{2}{n^{2}}\sum_{i,j=1}^{n}\xi_{i}\gamma_{j}-\frac{1}{n^{2}}\sum_{i,j=1}^{n}\gamma_{i}\gamma_{j}. \end{array}$$

Then, we have

(10) $$\begin{array}{c} \frac{\sqrt{n}}{\sqrt{v}}\left(V-\lambda_{H}\left(\theta \right)\right)=F+\frac{\sqrt{n}L}{\sqrt{v}}. \end{array}$$

Now, let $\kappa_{3}\left(F\right):=E\left[F^{3}\right],\;\kappa_{4}\left(F\right):=E\left[F^{4}\right]-3$ and it is equivalent to (8); let the process $F=I_{2}\left(g\right)$, where $g:=\frac{\sum_{i=1}^{n}\eta_{i}^{⊗2}}{\sqrt{nv}}$. By Nourdin–Peccati observations [24,25], one has for n≥1 the following.

$$\begin{array}{cc} \kappa_{3}\left(F\right)= & 2E\left[\left(F\right)(2I_{2}\left(g{\tilde{⊗}}_{1}g\right)+2∥g{∥}_{{ℋ}^{⊗}2}^{2})\right]\\ = & 8<g,g{\tilde{⊗}}_{1}g{>}_{{ℋ}^{⊗}2}^{2}=8{\langle g,g{⊗}_{1}g\rangle }_{{ℋ}^{⊗}2}^{2}\\ = & \frac{8}{{\left(nv\right)}^{\frac{3}{2}}}\sum_{i,j,k=1}^{n}{\langle \eta_{i},\eta_{k}\rangle }_{ℋ}{\langle \eta_{i},\eta_{j}\rangle }_{ℋ}{\langle \eta_{k},\eta_{j}\rangle }_{ℋ}\\ = & \frac{8}{{\left(nv\right)}^{\frac{3}{2}}}\sum_{i,j,k=1}^{n}E\left[\xi_{i}\xi_{k}\right]E\left[\xi_{i}\xi_{j}\right]E\left[\xi_{j}\xi_{k}\right] \end{array}$$

Therefore, using Young integral and the inequality of Hölder yields the following.

(11) $$\begin{array}{cc} |\kappa_{3}\left(F\right)|⊵ & \frac{1}{{\left(nv\right)}^{\frac{3}{2}}}\sum_{i,j,k=1}^{n}\left|r_{\xi }\left(i-k\right)r_{\xi }\left(i-j\right)r_{\xi }\left(j-k\right)\right|\\ ⊵ & \frac{n^{\frac{-1}{2}}}{{\left(v\right)}^{\frac{3}{2}}}{\left(\sum_{|k|<n}{\left|r_{\xi }\left(k\right)\right|}^{\frac{3}{2}}\right)}^{2}. \end{array}$$

Note that the symbol ⊵ means that the multiplicative universal constant has been excluded.

Similarly, for the fourth cumulant, we have the following.

(12) $$\begin{array}{cc} \kappa_{4}\left(F\right)= & \frac{1}{{\left(nv\right)}^{2}}\sum_{k,i,j,l=1}^{n}E\left[\xi_{k}\xi_{i}\right]E\left[\xi_{i}\xi_{j}\right]E\left[\xi_{j}\xi_{l}\right]E\left[\xi_{l}\xi_{k}\right]\\ \kappa_{4}\left(F\right)⊵ & \frac{1}{{\left(nv\right)}^{2}}\sum_{k,i,j,l=1}^{n}\left|r_{\xi }\left(k-l\right)r_{\xi }\left(i-j\right)r_{\xi }\left(j-l\right)r_{\xi }\left(l-k\right)\right|\\ ⊵ & \frac{n^{-1}}{{\left(v\right)}^{2}}{\left(\sum_{|k|<n}{\left|r_{\xi }\left(k\right)\right|}^{\frac{4}{3}}\right)}^{3}. \end{array}$$

Note that, there is no need to take the absolute value because the fourth-cumulant of the variable that lie sin the second chaos is positive.

Next, the total-variation distance between process *F* and the mean zero Normal distribution is provided by the following result.

**Theorem** **2.** *Suppose that $H\in (0,\frac{3}{4}]$ and let F be given by (8), then there is apositive constant C that depends only on F:*

$$\begin{array}{c} d_{TV}\left(F,\;N\left(0,\;1\right)\right)\leq C\max\left\{\frac{n^{\frac{-1}{2}}}{v^{\frac{3}{2}}}{\left(\sum_{|k|<n}{\left|k\right|}^{3H-3}\right)}^{2},\frac{n^{\frac{-1}{2}}}{v^{2}}{\left(\sum_{|k|<n}{\left|k\right|}^{\frac{8}{3}\left(H-1\right)}\right)}^{3}\right\}, \end{array}$$

*∀n≥1. Hence, the following estimates hold.*

$$\begin{array}{c} d_{TV}\left(F,N\left(0,1\right)\right)\leq \frac{C}{\max\{v^{2},v^{\frac{3}{2}}\}}\times \left\{\begin{array}{cc} n^{\frac{-1}{2}} & \mathrm{if}\;\;0<H<\frac{2}{3}\\ \frac{\log{\left(n\right)}^{2}}{\sqrt{n}} & \mathrm{if}\;\;\;H=\frac{2}{3}\\ n^{3(2H-\frac{3}{2})} & \mathrm{if}\;\;\frac{2}{3}<H<\frac{3}{4}\\ \log{\left(n\right)}^{\frac{-3}{2}} & \mathrm{if}\;\;\;H=\frac{3}{4}. \end{array}\right. \end{array}$$

**Proof.**  By Lemma A4 and estimation (11), we have the following:

- For $0<H<\frac{2}{3}$
  $$\begin{array}{c} \sum_{|k|<n}|r_{\xi }{\left(k\right)|}^{\frac{3}{2}}\leq C\sum_{|k|<n}{\left(|k\right|}^{2H-2}{)}^{\frac{3}{2}}=C\sum_{|k|<n}{\left|k\right|}^{3H-3}=C. \end{array}$$
- When $H=\frac{2}{3}$
  $$\begin{array}{c} \sum_{|k|<n}|r_{\xi }{\left(k\right)|}^{\frac{3}{2}}\leq C\sum_{|k|<n}{\left|k\right|}^{-1}\leq C\log\left(n\right). \end{array}$$
- For $\frac{2}{3}<H<\frac{3}{4}$
  $$\begin{array}{c} \sum_{|k|<n}|r_{\xi }{\left(k\right)|}^{\frac{3}{2}}\leq C\sum_{|k|<n}{\left|k\right|}^{3H-3}\leq Cn^{3H-2} \end{array}$$
- For $H=\frac{3}{4}$
  $$\begin{array}{c} \sum_{|k|<n}|r_{\xi }{\left(k\right)|}^{\frac{3}{2}}\leq C\sum_{|k|<n}{\left|k\right|}^{3H-3}\leq C\sqrt[{4}]{n}. \end{array}$$

Moreover, By Lemma A4, as well as the bound (12), one obtains the following approximation:

- For $0<H<\frac{5}{8}$
  $$\begin{array}{c} \sum_{|k|<n}|r_{\xi }{\left(k\right)|}^{\frac{4}{3}}\leq C\sum_{|k|<n}{\left(|k\right|}^{2H-2}{)}^{\frac{4}{3}}=C\sum_{|k|<n}{\left|k\right|}^{\frac{8}{3}H-\frac{8}{3}}=C. \end{array}$$
- When $H=\frac{5}{8}$
  $$\begin{array}{c} \sum_{|k|<n}|r_{\xi }{\left(k\right)|}^{\frac{4}{3}}\leq C\sum_{|k|<n}{\left|k\right|}^{-1}\leq C\log\left(n\right). \end{array}$$
- For $H\in (\frac{5}{8},\frac{3}{4})$
  $$\begin{array}{c} \sum_{|k|<n}|r_{\xi }{\left(k\right)|}^{\frac{4}{3}}\leq C\sum_{|k|<n}{\left|k\right|}^{\frac{8}{3}\left(H-1\right)}\leq Cn^{\frac{8}{3}H-\frac{5}{3}} \end{array}$$
- For $H=\frac{3}{4}$
  $$\begin{array}{c} \sum_{|k|<n}|r_{\xi }{\left(k\right)|}^{\frac{3}{4}}\leq C\sum_{|k|<n}{\left|k\right|}^{\frac{8}{3}\left(H-1\right)}\leq C\sqrt[{3}]{n}. \end{array}$$

Hence, it isproved.   □

**Remark** **1.** *In last theorem, we restrict the index H to be $H\leq \frac{3}{4}$ because this is the well known commencement for the validity limit of CLT. However, non-CLT holds for $H\in (\frac{3}{4},1)$, it has been shown in [26] that the speed of convergence in this case is slower than the case of $H\leq \frac{3}{4}$. Up to now, there is no known general framework that shows that the speeds obtained in non-CLT case are optimal or not; see the discussion on this point in Chapter 7 of [24].*

**Remark** **2.** *The estimates (11) and (12) can be given in a more accurate manner by letting C=0.4785 (see [27]). It is worth pointing out that, according to Esseen [28], the universal constant 0.4785 cannot be less than 0.40973.*

**Theorem** **3.** *Assume $H\in (0,\frac{3}{4}]$ is fixed and V is as defined in (4). Set $\sigma_{1,H}:=\sqrt{2\sum_{k\in Z}r_{\xi }{\left(k\right)}^{2}}$. Then, the following convergence holds.*

$$\begin{array}{c} \sqrt{n}\left(V-\lambda_{H}\left(\theta \right)\right)\;\;\;\underset{\to }{law}\;\;\;N\left(0,\sigma_{1,H}^{2}\right) \end{array}$$

**Proof.**  By virtue of lemma 9 in [2], it follows from expression (9), Theorem 2, Proposition A1 as well as Lemma A5 that the following is the case:

- When $0<H<\frac{2}{3}$
  $$\begin{array}{c} d_{W}\left(\frac{\sqrt{n}}{\sigma_{1,H}}\left(V-\lambda_{H}\left(\theta \right)\right),N\left(0,1\right)\right)\leq C(\sqrt{n}{∥L∥}_{L^{1}\left(\Omega \right)}+|v-\sigma_{1,H}^{2}\left|+n^{\frac{-1}{2}}\right) \end{array}$$
- For $H=\frac{2}{3}$
  $$\begin{array}{c} d_{W}\left(\frac{\sqrt{n}}{\sigma_{1,H}}\left(V-\lambda_{H}\left(\theta \right)\right),N\left(0,1\right)\right)\leq C(\sqrt{n}{∥L∥}_{L^{1}\left(\Omega \right)}+|v-\sigma_{1,H}^{2}\left|+\frac{\log{\left(n\right)}^{2}}{\sqrt{n}}\right) \end{array}$$
- For $H\in (\frac{2}{3},\frac{3}{4})$
  $$\begin{array}{c} d_{W}\left(\frac{\sqrt{n}}{\sigma_{1,H}}\left(V-\lambda_{H}\left(\theta \right)\right),N\left(0,1\right)\right)\leq C(\sqrt{n}{∥L∥}_{L^{1}\left(\Omega \right)}+|v-\sigma_{1,H}^{2}\left|+n^{3(2H-\frac{3}{2})}\right) \end{array}$$
- For $H=\frac{3}{4}$
  $$\begin{array}{cc} \; & d_{W}\left(\frac{\sqrt{n}}{\sigma_{1,\frac{3}{4}}\log{\left(n\right)}^{\frac{1}{2}}}\left(V-\lambda_{\frac{3}{4}}\left(\theta \right)\right),N\left(0,1\right)\right)\\ \; & \leq C(\frac{\sqrt{n}}{\log{\left(n\right)}^{\frac{1}{2}}}∥L{∥}_{L^{1}\left(\Omega \right)}+|\frac{v}{\log{\left(n\right)}^{\frac{1}{2}}}-\sigma_{1,\frac{3}{4}}^{2}|+\log{\left(n\right)}^{\frac{-3}{2}}). \end{array}$$

Thus, the theorem is proved.   □

We are now prepared to prove that the theorem investigates the rate of normal convergence of estimator $\hat{\theta }$ toward parameter θ.

**Theorem** **4.** *Let $x={\left(x_{t}\right)}_{t\geq 0}$ be given by (1) and (2), where $S^{H}$ is a sfBm and parameter $H\in (0,\frac{3}{4}]$. The following convergence takes place as n⟶∞:*

- *If $0<H<\frac{1}{2}$*
$$\begin{array}{cc} d_{W}\left(\frac{8H^{2}\Gamma \left(2H\right)}{\sigma_{1,H}\theta^{2H+1}}\sqrt{n}\left(\hat{\theta }-\theta \right),N\left(0,1\right)\right) & \leq Cn^{\frac{-1}{2}} \end{array}$$
- *If $\frac{1}{2}<H<\frac{3}{4}$*
$$\begin{array}{cc} d_{W}\left(\frac{8H^{2}\Gamma \left(2H\right)}{\sigma_{1,H}\theta^{2H+1}}\sqrt{n}\left(\hat{\theta }-\theta \right),N\left(0,1\right)\right) & \leq Cn^{2H-\frac{3}{2}} \end{array}$$
- *If $H=\frac{3}{4}$*
$$\begin{array}{c} d_{W}\left(\frac{6\Gamma (\frac{3}{2})}{\theta^{\left(\frac{1}{2}\right)}\log{\left(n\right)}^{\frac{1}{2}}}\sqrt{n}\left(\hat{\theta }-\theta \right),N\left(0,1\right)\right)\leq \frac{C}{\sqrt{\log(n)}}. \end{array}$$

**Proof.**  The result follows by applying Theorem 3 and using the technique used in Section 5.2.2 of [2] for $\hat{\theta }=\lambda_{H}^{-1}\left(V\right)$. Note that we make use of $\sigma_{1,\frac{3}{4}}=\frac{3}{4}\theta^{-2}$; see Proposition A1.   □

In what follows, the convergence in the law of $\hat{\mu }$ to μ is shown by the next theorem.

**Theorem** **5.** *Consider the process $x={\left(x_{t}\right)}_{t\geq 0}$ defined by (1) and (2); if 0<H<1, then there is a function denoted by ϕ such that the following is the case:*

(13) $$\begin{array}{c} \phi \left(\hat{\mu }-\mu \right)\;\;\;\underset{\to }{law}\;\;\;N\left(0,\sigma_{2,H}^{2}\right) \end{array}$$

*where $\sigma_{2,H}^{2}:=2\sum_{k\in N^{+}}r_{\xi }\left(k\right)+2\Gamma \left(2H+1\right)\theta^{-2H}$, when $0<H\leq \frac{1}{2}$, $N^{+}=N\backslash \left\{0\right\}$ and $\sigma_{2,H}^{2}:=\theta^{-2}$, when $\frac{1}{2}<H<1$.*

**Proof.**  For n≥1, we define the following:

$$\begin{array}{c} \phi :=\left\{\begin{array}{cc} n^{\frac{1}{2}} & \mathrm{if}\;\;0<H\leq \frac{1}{2}\\ n^{-(H-1)} & \mathrm{if}\;\;\;\frac{1}{2}<H<1 \end{array}\right. \end{array}$$

and let $K:=\frac{\phi }{n}\sum_{i=1}^{n}e^{-\theta (i-\cdot )}1_{\left[0,i](\cdot \right)}$.

From Equation (5) and the definition of $\hat{\mu }$, we have the following.

$$\begin{array}{cc} \phi (\hat{\mu }-\mu )= & \frac{\phi }{n}\sum_{i=1}^{n}\int_{0}^{i}e^{-\theta (i-s)}dS_{s}^{H}-\frac{\phi }{n}\sum_{i=1}^{n}e^{-\theta i}\\ = & I_{1}\left(K\right)-\frac{\phi }{n}\sum_{i=1}^{n}e^{-\theta i} \end{array}$$

Hence, $\phi (\hat{\mu }-\mu )$ represents a Gaussian process.

Let $\beta :=\max\{∥I_{1}\left(K\right){∥}_{L^{2}\left(\Omega \right)},\sigma_{2,H}^{2}\}$. Then, by applying Lemma A6, for $0<H\leq \frac{1}{2}$, we obtain the following.

$$\begin{array}{cc} d_{W}\left(I_{1}\left(K\right),N\left(0,\sigma_{2,H}^{2}\right)\right)\leq & \frac{\sqrt{2/\pi }}{\beta }|E\left[I_{1}{\left(K\right)}^{2}\right]-\sigma_{2,H}^{2}|\\ \leq & \frac{C\sqrt{2/\pi }\;n^{2H-1}}{\beta } \end{array}$$

Now, it follows by lemma 9 in [2] that the following is the case.

$$\begin{array}{c} d_{W}\left(\phi \left(\hat{\mu }-\mu \right),N\left(0,\sigma_{2,H}^{2}\right)\right)\leq \frac{C\sqrt{2/\pi }\;n^{2H-1}}{\beta }+Cn^{-1}. \end{array}$$

Similarly, for $\frac{1}{2}<H<1$, we conclude the following.

$$\begin{array}{c} d_{W}\left(I_{1}\left(K\right),N\left(0,\sigma_{2,H}^{2}\right)\right)\leq \frac{\sqrt{2/\pi }}{\beta }|E\left[I_{1}{\left(K\right)}^{2}\right]-\theta^{-2}|⟶0,\;n\to +\infty \end{array}$$

Finally, as n→+∞, we have the following:

$$\begin{array}{c} d_{W}\left(\phi \left(\hat{\mu }-\mu \right),N\left(0,\theta^{-2}\right)\right)\leq Cn^{-1} \end{array}$$

which complete the proof.   □

## 5. Numerical Illustrations

Simulation of the sfBm sample paths [29] is essential for studying SDEs driven by these type of processes. In fact, generating the sample paths of sfBm allows us to investigate the approximation solution and parameter estimation for sfBm-driven SDE. Until now, there are a few studies devoted to simulate sfBm; some of these monographs, such as [30] have used the fact that sfBm can be given by means of fBm, namely the following.

$$S_{t}^{H}=\frac{1}{\sqrt{2}}\left(W_{t}^{H}+W_{-t}^{H}\right);\;t\geq 0,$$

However, Brownian motion has been implemented by some scholars to generate the sample paths of sfBm. Different methods are used to construct sfBm with Bm; for instance, in [31], the following equation is applied:

$$S_{t}^{H}=\frac{\sqrt{\Gamma (1+2H)\sin(\pi H)}}{\pi }\int_{0}^{t}n_{H}\left(t,s\right)dW_{s},$$

where the following is the case.

$$\begin{array}{c} n_{H}\left(t,s\right)=\frac{\sqrt{\pi }s^{3/2-H}}{2^{H}\Gamma \left(H+1/2\right)}\left[\frac{{\left(t^{2}-s^{2}\right)}^{H-1/2}}{t}+\int_{s}^{t}\frac{{\left(x^{2}-s^{2}\right)}^{H-1/2}}{x^{2}}dx\right];\;0<s<t<\infty . \end{array}$$

The sample paths of sfBm can be approximated by Brownian motion [32]:

(14) $$\begin{array}{c} S_{t}^{H}=K_{H}^{-1}\int_{R}{\left[t-s\right]}_{+}^{H-1/2}+{\left[-t-s\right]}_{+}^{H-1/2}-2{\left[-s\right]}_{+}^{H-1/2}dW_{s}, \end{array}$$

where $K_{H}=\sqrt{2\int_{R}{\left|{\left[1-s\right]}_{+}^{H-1/2}-{\left[-s\right]}_{+}^{H-1/2}\right|}^{2}ds+\frac{1}{2H}}$ represents the normalization constant.

It is worth mentioning that we have discovered that in comparison with other methods, the last formula is more obedient and implementable in the computation of sfBm.

In the following, we simulate sfBm through the procedures below:

1. Set sample size to be N∈N and the time span is *T*;
2. Choose two different values of parameter *H*;
3. Select a mesh size m∈N and a cut–off index M∈N;
4. Evaluate the normalization constant;
  $$\begin{array}{c} K_{H}=\frac{1}{m}\sqrt{2\sum_{j=1}^{mM}{\left|{\left[j/m\right]}_{+}^{H-1/2}-{\left[\left(j/m\right)-1\right]}_{+}^{H-1/2}\right|}^{2}+\frac{1}{2H}}. \end{array}$$
5. Using its increments, write the sequence SH(N) as follows;
  (15) $$\begin{array}{c} S^{H}\left(N\right)=\sum_{k=1}^{N}S^{H}\left(k\right)-S^{H}\left(k-1\right):=\sum_{k=1}^{N}{\tilde{S}}^{H}\left(k\right), \end{array}$$
6. Compute the increments of ${\tilde{S}}^{H}$ by applying the Riemann sum.
  $$\begin{array}{cc} {\tilde{S}}^{H}\left(k\right)= & K_{H}^{-1}\sum_{j=1}^{mM}\left({\left[j/m\right]}_{+}^{H-1/2}-{\left[\left(j/m\right)-1\right]}_{+}^{H-1/2}\right)\\ \; & +({\left[\left(j/m\right)-2k\right]}_{+}^{H-1/2}-{\left[\left(j/m\right)-2k+1\right]}_{+}^{H-1/2})▵W\left(mk-j\right), \end{array}$$
  $$\begin{array}{c} ▵W\left(j\right):=W\left(\frac{j+1}{m}\right)-W\left(\frac{j}{m}\right)=\sqrt{\frac{j+1}{m}-\frac{j}{m}}\times N\left(0,1\right). \end{array}$$

**Figure 1:**
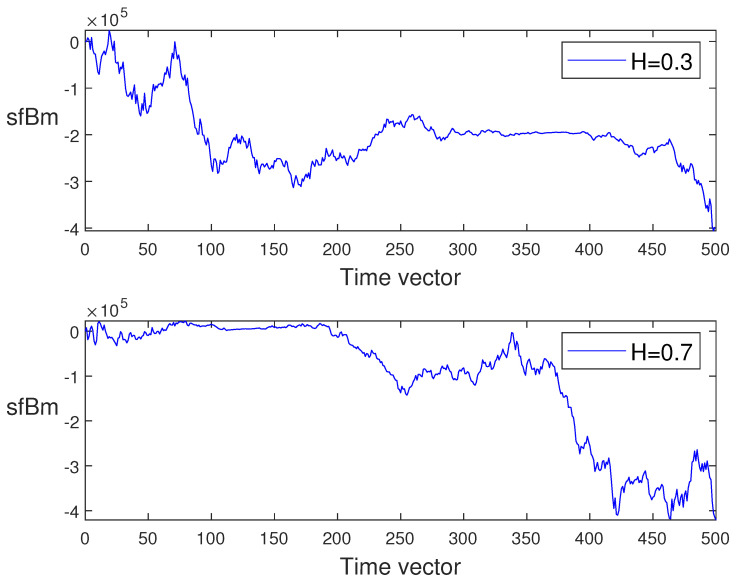
The sample paths of sub-fractional Brownian motions.

**Figure 2:**
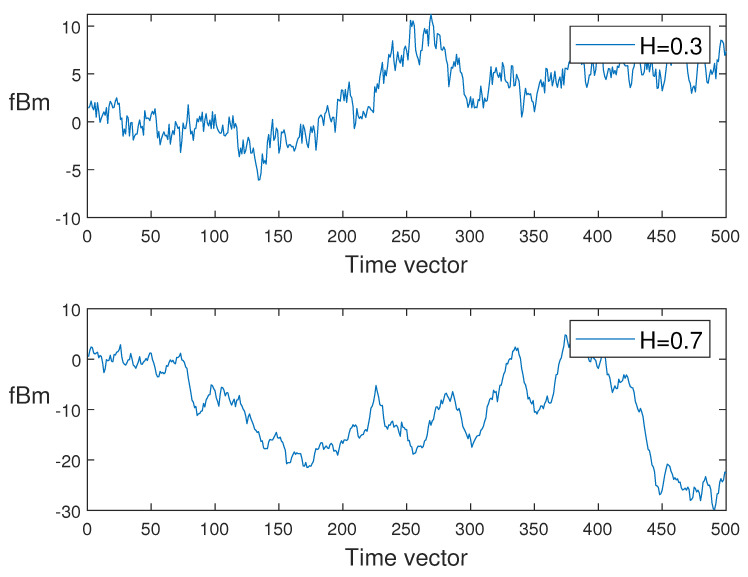
Discretized fractional Brownian motion path.

In what follows, for different values of *H*, we generate the sample paths of sfBm in Figure 1. Moreover, to compare the trajectories of sfBm and fBm, the paths of fBm have been simulated with two values of H={0.3,0.7}; see Figure 2.

**Figure 3:**
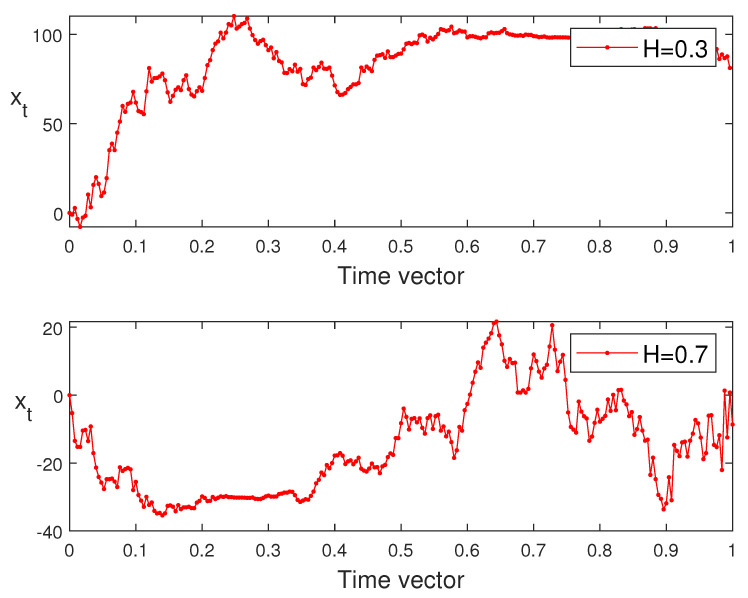
The sub-fractional Vasicek process simulation.

Next, in Figure 3, we generate the sample paths of sub-fractional Vasicek process defined by Equations (8) and (9) for various values of *H* = 0.3, 0.7, θ = 0.009, 0.003, and μ = 0.004, 0.008.

From Simulation 4.3, we see clearly that the characterization of the sample path of sub-fractional Vasicek process can be determined by the values of *H*. In other words, we obtain almost smooth sample paths of $x_{t}$ in the case of the small Hurst parameter: *H* = 0.3, especially when t⟶T. On the other hand, large values of *H* (in this case, we choose *H* to be 0.7) make the sample path of $x_{t}$ fluctuate more wildly particularly when *t* tends to its final value *T*. However, by changing the parameters θ and μ, weobtainget different values of mean and standard deviation for $x_{t}$ such that in the case of θ = 0.009 and μ = 0.004; we obtain smaller mean and bigger standard deviation than the second case.

## 6. Conclusions

In this paper, we present a new method for estimating unknown parameters in the Vasicek-type model observed throughout a period of discrete time. Furthermore, we demonstrated that the calculated parameters and the original parameters are similar enough using Malliavin calculus and Nordin–Peccati analysis. The proposed estimators have been demonstrated to be highly consistent and asymptotically normal. Meanwhile, numerical simulations of sub-fractional Brownian motion and sub-fractional Vasicek-type process have been provided for various values of the Hurst index *H*.

## Data Availability

No data were used to support this work.

## Appendix A

**Lemma** **A1.**
*Let $\left\{S_{t}^{H},t\geq 0\right\}$ be an sfBm with H∈(0,1]. Let $t_{0}\in \left[0,\infty \right)$ and λ>0. Then, we have for all $t>t_{0}$ the following.*

$$\begin{array}{c} \int_{t_{0}}^{t}e^{\lambda u}dS_{u}^{H}=e^{\lambda t}S_{t}^{H}-e^{\lambda t_{0}}S_{t_{0}}^{H}-\lambda \int_{t_{0}}^{t}e^{\lambda u}S_{u}^{H}du. \end{array}$$

**Proof.** We conclude the result by using the same argument as in the proof of proposition A.1 in [33].   □

**Lemma** **A2.**
*Let H∈(0,1)\{1/2} and consider the stochastic process ξ defined in (6), then we have the following.*

$$\begin{array}{c} E\left[\xi_{0}^{2}\right]=2\Gamma \left(2H+1\right)\theta^{-2H}. \end{array}$$

**Proof.** Suppose that we have two stochastic processes $\xi^{\lambda_{1}}$, $\xi^{\lambda_{2}}$ with positive integers $\lambda_{1},\lambda_{2}$. Then, the expression (6) and Lemma A1 allow us to write the following:

$$\begin{array}{cc} E[\xi_{0}^{\lambda_{1}}\xi_{0}^{\lambda_{2}}]= & E\int_{-\infty }^{0}\int_{-\infty }^{0}e^{\lambda_{1}u}e^{\lambda_{2}v}dS_{u}^{H}dS_{v}^{H}\\ = & \lambda_{1}\lambda_{2}\int_{0}^{\infty }\int_{0}^{\infty }e^{\lambda_{1}u}e^{\lambda_{2}v}E\left[S_{u}^{H}S_{v}^{H}\right]dudv\\ = & \lambda_{1}\lambda_{2}\int_{0}^{\infty }\int_{0}^{\infty }e^{\lambda_{1}u}e^{\lambda_{2}v}[u^{2H}+v^{2H}-\frac{1}{2}{(\left(v+u\right)}^{2H}{+|v-u|}^{2H})]dudv\\ = & \frac{2\Gamma (2H+1)}{\lambda_{1}+\lambda_{2}}\left[\lambda_{1}^{1-2H}+\lambda_{2}^{1-2H}\right]. \end{array}$$

which complete the proof.   □

**Lemma** **A3.**
*Suppose that H∈(0,1)\{1/2} and consider the following constant −∞≤a<b≤c<d<∞, then*

$$\begin{array}{cc} E\left[\int_{a}^{b}e^{\lambda u}dS_{u}^{H}\int_{c}^{d}e^{\lambda v}dS_{v}^{H}\right]= & H(2H-1)\times \\ \; & \int_{a}^{b}e^{\lambda u}\left(\int_{c}^{d}e^{\lambda v}\left[{\left(v+u\right)}^{2H-2}+{\left(v-u\right)}^{2H-2}\right]dv\right)du. \end{array}$$

**Proof.** First, by using Lemma A1 and suppose that b=0=c; then we have the following.

$$\begin{array}{cc} \Sigma := & E\left[\int_{a}^{0}e^{\lambda u}dS_{u}^{H}\int_{0}^{d}e^{\lambda v}dS_{v}^{H}\right]\\ = & E\left[\left(-e^{\lambda a}S_{a}^{H}-\lambda \int_{a}^{0}e^{\lambda u}S_{u}^{H}du\right)\left(e^{\lambda d}S_{d}^{H}-\lambda \int_{0}^{d}e^{\lambda v}S_{v}^{H}dv\right)\right]\\ = & -e^{\lambda a}e^{\lambda d}E\left[S_{d}^{H}S_{a}^{H}\right]+\lambda e^{\lambda a}\int_{0}^{d}e^{\lambda v}E\left[S_{a}^{H}S_{v}^{H}\right]dv\\ - & \lambda e^{\lambda d}\int_{a}^{0}e^{\lambda u}E\left[S_{u}^{H}S_{d}^{H}\right]du+\lambda^{2}\int_{0}^{d}e^{\lambda v}\left(\int_{a}^{0}e^{\lambda u}E\left[S_{u}^{H}S_{v}^{H}\right]du\right)dv\\ = & -e^{\lambda a}e^{\lambda d}\left({\left(-a\right)}^{2H}+d^{2H}-\frac{1}{2}\left[{\left(d+a\right)}^{2H}+{\left(d-a\right)}^{2H}\right]\right)\\ + & \lambda e^{\lambda a}\int_{0}^{d}e^{\lambda v}{(\left(-a\right)}^{2H}+v^{2H}-\frac{1}{2}\left[{\left(v+a\right)}^{2H}+{\left(v-a\right)}^{2H}\right]dv\\ - & \lambda e^{\lambda d}\int_{a}^{0}e^{\lambda u}{(\left(-u\right)}^{2H}+d^{2H}-\frac{1}{2}\left[{\left(d+u\right)}^{2H}+{\left(d-u\right)}^{2H}\right]du\\ + & \lambda^{2}\int_{0}^{d}e^{\lambda v}\left(\int_{a}^{0}e^{\lambda u}{(\left(-u\right)}^{2H}+v^{2H}-\frac{1}{2}\left[{\left(v+u\right)}^{2H}+{\left(v-u\right)}^{2H}\right]du\right)dv \end{array}$$

By applying integration by parts with respect to *u* first and then with respect to *v*, we conclude the following.

$$\begin{array}{cc} \Sigma = & -2He^{\lambda d}\int_{a}^{0}e^{\lambda u}\left({\left(-u\right)}^{2H-1}-\frac{1}{2}\left[{\left(d+u\right)}^{2H-1}+{\left(d-u\right)}^{2H-1}\right]\right)du\\ + & 2H\lambda \int_{0}^{d}e^{\lambda v}\left(\int_{a}^{0}e^{\lambda u}\left({\left(-u\right)}^{2H-1}-\frac{1}{2}\left[{\left(v+u\right)}^{2H-1}+{\left(v-u\right)}^{2H-1}\right]\right)du\right)dv\\ = & H\left(2H-1\right)\int_{a}^{0}e^{\lambda u}\left(\int_{0}^{d}e^{\lambda v}\left[{\left(v+u\right)}^{2H-2}+{\left(v-u\right)}^{2H-2}\right]dv\right)du \end{array}$$

In what follows, we let 0<c,b=0, and using the above computation, one obtains the following.

$$\begin{array}{cc} \; & E\left[\int_{a}^{0}e^{\lambda u}dS_{u}^{H}\int_{c}^{d}e^{\lambda v}dS_{v}^{H}\right]=E\left[\int_{a}^{0}e^{\lambda u}dS_{u}^{H}\int_{0}^{d}e^{\lambda v}dS_{v}^{H}-\int_{a}^{0}e^{\lambda u}dS_{u}^{H}\int_{0}^{c}e^{\lambda v}dS_{v}^{H}\right]\\ = & H\left(2H-1\right)[\int_{a}^{0}e^{\lambda u}\left(\int_{0}^{d}e^{\lambda v}\left[{\left(v+u\right)}^{2H-2}+{\left(v-u\right)}^{2H-2}\right]dv\right)du\\ - & \int_{a}^{0}e^{\lambda u}\left(\int_{0}^{c}e^{\lambda v}\left[{\left(v+u\right)}^{2H-2}+{\left(v-u\right)}^{2H-2}\right]dv\right)du]\\ = & H\left(2H-1\right)\int_{a}^{0}e^{\lambda u}\left(\int_{c}^{d}e^{\lambda v}\left[{\left(v+u\right)}^{2H-2}+{\left(v-u\right)}^{2H-2}\right]dv\right)du. \end{array}$$

Generally, in the case of −∞≤a<b≤c<d<∞, we have $\tilde{S_{t}^{H}}:=S_{t+b}^{H}-S_{b}^{H};\;t\in R$ represents a sub-fractional Brownian motion too. Therefore, the following is the case.

$$\begin{array}{cc} E\left[\int_{a}^{b}e^{\lambda u}dS_{u}^{H}\int_{c}^{d}e^{\lambda v}dS_{v}^{H}\right]= & E\left[\int_{a-b}^{0}e^{\lambda (w+b)}d{\tilde{S}}_{w}^{H}\int_{c-b}^{d-b}e^{\lambda (x+b)}d{\tilde{S}}_{x}^{H}\right]\\ = & H\left(2H-1\right)\int_{a}^{b}e^{\lambda u}\left(\int_{c}^{d}e^{\lambda v}\left[{\left(v+u\right)}^{2H-2}+{\left(v-u\right)}^{2H-2}\right]dv\right)du. \end{array}$$

This completes the proof.   □

**Lemma** **A4.**
*Assume that H∈(0,1)\{1/2} and consider the constants $\lambda_{1},\lambda_{2}>0$. Thus, for large |t|, we have the following:*

$$\begin{array}{c} E\left[\xi_{0}^{\lambda_{1}}\xi_{t}^{\lambda_{2}}\right]∼\frac{2H(2H-1)}{\lambda_{1}\lambda_{2}}{\left|t\right|}^{2H-2}. \end{array}$$

*where we mean by a∼b that there are two constants c,C>0 such that cb≤a≤Cb.*

**Proof.** Thanks to Lemma A1, one can write the following:

$$\begin{array}{cc} E[\xi_{0}^{\lambda_{1}}\xi_{t}^{\lambda_{2}}]= & e^{-\lambda_{2}t}E\left[\int_{-\infty }^{0}e^{\lambda_{1}u}dS_{u}^{H}\int_{-\infty }^{t}e^{\lambda_{2}v}dS_{v}^{H}\right]\\ = & e^{-\lambda_{2}t}E\left[\int_{-\infty }^{0}e^{\lambda_{1}u}dS_{u}^{H}\int_{-\infty }^{\epsilon t}e^{\lambda_{2}v}dS_{v}^{H}\right]+e^{-\lambda_{2}t}E\left[\int_{-\infty }^{0}e^{\lambda_{1}u}dS_{u}^{H}\int_{\epsilon t}^{t}e^{\lambda_{2}v}dS_{v}^{H}\right]\\ = & :L_{1}+L_{2} \end{array}$$

where ϵ∈(0,1). It is obvious that $|L_{1}|=O\left(e^{-\lambda_{2}t}\right)$. On the other hand, using the calculus of Lemma A3, we obtain the following.

$$\begin{array}{cc} L_{2}= & H\left(2H-1\right)e^{-\lambda_{2}t}\int_{-\infty }^{0}e^{\lambda_{1}u}\left(\int_{\epsilon t}^{t}e^{\lambda_{2}v}\left[{\left(v-u\right)}^{2H-2}+{\left(v+u\right)}^{2H-2}\right]dv\right)du\\ = & H\left(2H-1\right)e^{-\lambda_{2}t}\int_{-\infty }^{0}e^{\lambda_{1}u}\left(\int_{\epsilon t}^{t}e^{\lambda_{2}v}{\left(v-u\right)}^{2H-2}dv\right)du\\ + & H\left(2H-1\right)e^{-\lambda_{2}t}\int_{-\infty }^{0}e^{\lambda_{1}u}\left(\int_{\epsilon t}^{t}e^{\lambda_{2}v}{\left(v+u\right)}^{2H-2}dv\right)du\\ =: & L_{21}+L_{22}. \end{array}$$

Now, by applying change of variables for the portion $L_{21}$ and using the technique of partial integration, one obtains the following:

$$\begin{array}{cc} L_{21}= & \frac{H(2H-1)}{\lambda_{1}+\lambda_{2}}[\int_{t}^{\infty }e^{-\lambda_{1}\left(w-t\right)}w^{2H-2}dw\\ + & \int_{\epsilon t}^{t}e^{-\lambda_{2}\left(t-w\right)}w^{2H-2}dw+e^{-\lambda_{2}t\left(1-\epsilon \right)}\int_{\epsilon t}^{\infty }e^{-\lambda_{1}\left(w-\epsilon t\right)}w^{2H-2}dw]\\ = & \frac{H(2H-1)}{\lambda_{1}+\lambda_{2}}(\frac{t^{2H-2}}{\lambda_{1}}+\frac{2H-2}{\lambda_{1}}\int_{t}^{\infty }e^{-\lambda_{1}\left(w-t\right)}w^{2H-3}dw\\ + & \frac{t^{2H-2}}{\lambda_{2}}-\frac{{\left(\epsilon t\right)}^{2H-2}}{\lambda_{2}}e^{-\lambda_{2}t\left(1-\epsilon \right)}-\frac{2H-2}{\lambda_{2}}\int_{\epsilon t}^{t}e^{-\lambda_{2}\left(t-w\right)}w^{2H-3}dw\\ + & e^{-\lambda_{2}t\left(1-\epsilon \right)}\int_{\epsilon t}^{\infty }e^{-\lambda_{1}\left(w-\epsilon t\right)}w^{2H-2}dw)\\ = & \frac{H(2H-1)}{\lambda_{1}\lambda_{2}}t^{2H-2}+o\left(t^{2H-2}\right)\\ ∼ & \frac{H(2H-1)}{\lambda_{1}\lambda_{2}}t^{2H-2} \end{array}$$

where

- $\int_{t}^{\infty }e^{-\lambda_{1}\left(w-t\right)}w^{2H-3}dw\leq t^{-1}\int_{0}^{\infty }e^{-\lambda_{1}y}dy⟶0$ as t⟶∞;
- $t^{2-2H}\int_{\epsilon t}^{t}e^{-\lambda_{2}\left(t-w\right)}w^{2H-3}dw\leq \epsilon^{2H-3}t^{-1}\int_{\epsilon t}^{t}e^{-\lambda_{2}\left(t-w\right)}dw=\epsilon^{2H-3}t^{-1}\int_{0}^{t(1-\epsilon )}e^{-\lambda_{2}y}dy⟶0$ as t⟶∞;
- $t^{2-2H}e^{-\lambda_{2}t\left(1-\epsilon \right)}⟶0$ as t⟶∞.

By the same argument, we deduce that $L_{22}∼\frac{H(2H-1)}{\lambda_{1}\lambda_{2}}t^{2H-2}$. Therefore, the result is obtained.   □

**Proposition** **A1.**
*Let the process ξ as in (6); under the assumptions of Theorem 2, ∀n≥1, the following statements are true:*

- *When $0<H<\frac{1}{2}$*
  $$\begin{array}{c} |v-\sigma_{1,H}^{2}|\leq Cn^{-1} \end{array}$$
- *When $\frac{1}{2}<H<\frac{3}{4}$*
  $$\begin{array}{c} |v-\sigma_{1,H}^{2}|\leq Cn^{4H-3}. \end{array}$$
  *where $\sigma_{1,H}=\sqrt{2\sum_{k=1}^{n}r_{\xi }^{2}\left(k\right)}$*.
- *Finally, when $H=\frac{3}{4}$, we have the following:*
  $$\begin{array}{c} |\frac{v}{\log(n)}-\sigma_{1,\frac{3}{4}}^{2}{|\leq C\log\left(n\right)}^{-1}. \end{array}$$
  *where $\sigma_{1,\frac{3}{4}}=\frac{3}{4}\theta^{-2}$.*

**Proof.** By definition of variable *v*, one obtains the following, as n→∞.

$$\begin{array}{cc} v=\frac{2}{n}\sum_{i,k}{\left(E\left[\xi_{i}\xi_{k}\right]\right)}^{2}= & \frac{4}{n}\sum_{k=1}^{n}r_{\xi }^{2}\left(k\right)\left(n-k\right)+2r_{\xi }^{2}\left(0\right). \end{array}$$

It follows by Lemma A4 that the following is the case:

$$\begin{array}{c} |v-\sigma_{1,H}^{2}|\leq \frac{2}{n}\sum_{k=1}^{n-1}k^{4H-3}+2\sum_{k=n}^{\infty }k^{4H-4} \end{array}$$

and if $H\in (0,\frac{1}{2})$, one has the following.

$$\begin{array}{c} |v-\sigma_{1,H}^{2}|\leq Cn^{-1} \end{array}$$

On the other hand, when $H\in (\frac{1}{2},\frac{3}{4})$, we obviously have the following.

$$\begin{array}{c} |v-\sigma_{1,H}^{2}|\leq Cn^{4H-3}. \end{array}$$

Moreover, if $H=\frac{3}{4}$, we have $\sum_{k=1}^{n}r_{\xi }^{2}\left(k\right)∼\frac{9}{64}\log\left(n\right)$ for large *n*.

$$\begin{array}{c} |\frac{v}{\log(n)}-\frac{9}{16\theta^{4}}{|\leq C\;\log\left(n\right)}^{-1} \end{array}$$

Hence, the proof is completed.   □

**Lemma** **A5.**
*Let parameter $H\in (0,\frac{3}{4}]$ and the random process L be given by (9); then we have the following.*

$$\begin{array}{c} \sqrt{n}E\left|L\right|⊴\left\{\begin{array}{cc} n^{\frac{-1}{2}} & \mathrm{if}\;\;0<H<\frac{1}{2}\\ n^{2H-\frac{3}{2}} & \mathrm{if}\;\;\;\frac{1}{2}<H<\frac{3}{4} \end{array}\right. \end{array}$$

*If $H=\frac{3}{4}$, then the following is the case.*

$$\begin{array}{c} \frac{\sqrt{n}}{\sqrt{log(n)}}E\left|L\right|⊴log{\left(n\right)}^{-1/2}. \end{array}$$

**Proof.** The result follows from definition ofthe process *L* in (9), Lemma A4, and the calculus of Lemma 5.3 in [34].   □

**Lemma** **A6.**
*Under the assumptions and notions of Theorem 5, the following cases are fulfilling:*

- *If $H\in (0,\frac{1}{2}]$, then the following is the case;*
  $$\begin{array}{c} \chi :=|E\left[I_{1}{\left(K\right)}^{2}\right]-\sigma_{2,H}^{2}|\leq Cn^{2H-1} \end{array}$$
- *If $H\in (\frac{1}{2},1)$, then the following is the case:*
  (A1)
  $$\begin{array}{c} E\left[I_{1}{\left(K\right)}^{2}\right]\;\to \;\theta^{-2} \end{array}$$
  *such that $I_{1}\left(K\right):=\frac{\phi }{n}\left(\sum_{i=1}^{n}\xi_{i}-\sum_{i=1}^{n}e^{-\theta i}\xi_{0}\right)$ and $\sigma_{2,H}^{2}=2\sum_{k\in N^{+}}r_{\xi }\left(k\right)+r_{\xi }\left(0\right)$.*

**Proof.** In order to estimate χ, first, let us compute $E[I_{1}{\left(K\right)}^{2}]$. By the definition of ϕ (Theorem 5), we obtain the following.

$$\begin{array}{cc} E[I_{1}{\left(K\right)}^{2}]= & \frac{1}{n}\sum_{i,j=1}^{n}E\left[\left(\xi_{i}-e^{-\theta i}\xi_{0}\right)\left(\xi_{j}-e^{-\theta j}\xi_{0}\right)\right]\\ = & \frac{1}{n}\sum_{i,j=1}^{n}E\left[\xi_{i}\xi_{j}\right]-\frac{2}{n}\sum_{i,j=1}^{n}e^{-\theta i}E\left[\xi_{j}\xi_{0}\right]+\frac{1}{n}{\left(\sum_{i=1}^{n}e^{-\theta i}\right)}^{2}E{\left[\xi_{0}\right]}^{2}\\ = & r_{\xi }\left(0\right)+\frac{2}{n}\sum_{i=1}^{n-1}\sum_{j=i+1}^{n}r_{\xi }\left(j-i\right)-\frac{2}{n}\sum_{i,j=1}^{n}r_{\xi }\left(j\right)e^{-\theta i}+\frac{r_{\xi }\left(0\right)}{n}{\left(\sum_{i=1}^{n}e^{-\theta i}\right)}^{2} \end{array}$$

Thus, we have the following:

$$\begin{array}{cc} \chi & \leq |\frac{2}{n}\sum_{i,j=1}^{n}r_{\xi }\left(j-i\right)-2\sum_{k\in N^{+}}r_{\xi }\left(k\right)|+|\frac{2}{n}\sum_{i,j=1}^{n}r_{\xi }\left(j\right)e^{-\theta i}+\frac{r_{\xi }\left(0\right)}{n}{\left(\sum_{i=1}^{n}e^{-\theta i}\right)}^{2}|\\ \; & \leq |\frac{2}{n}\sum_{k=1}^{n}\left(n-k\right)r_{\xi }\left(k\right)-2\sum_{k\in N^{+}}r_{\xi }\left(k\right)|+|\frac{2}{n}\sum_{j=1}^{n}r_{\xi }\left(j\right)\sum_{i=1}^{n}e^{-\theta i}+\frac{r_{\xi }\left(0\right)}{n}{\left(\sum_{i=1}^{n}e^{-\theta i}\right)}^{2}|\\ \; & \leq |\frac{2}{n}\sum_{k=1}^{n-1}kr_{\xi }\left(k\right)|+|2\sum_{k=n}^{\infty }r_{\xi }\left(k\right)|+|\frac{2}{n}\sum_{j=1}^{n}r_{\xi }\left(j\right)\sum_{i=1}^{n}e^{-\theta i}\left|+\right|\frac{4H\Gamma (2H)}{n\theta^{2H}}{\left(\sum_{i=1}^{n}e^{-\theta i}\right)}^{2}|\\ \; & \leq C\left(\frac{1}{n}\sum_{k=1}^{n-1}k^{2H-1}+\sum_{k=n}^{\infty }k^{2H-2}+n^{-1}+n^{-1}\right)\\ \; & \leq Cn^{2H-1} \end{array}$$

where $H<\frac{1}{2}$ and $\sum_{k}r_{\xi }\left(k\right)<\infty$. Thus, the first part of the lemma is shown. Now, to prove the second claim of the lemma, we have $I_{1}\left(K\right):=\frac{1}{n^{H}}\sum_{i=1}^{n}\left(\xi_{i}-e^{-\theta i}\xi_{0}\right)$, and similarly to the first case, we have the following.

$$\begin{array}{cc} E[I_{1}{\left(K\right)}^{2}]= & \frac{1}{n^{2H}}\sum_{i,j=1}^{n}E\left[\left(\xi_{i}-e^{-\theta i}\xi_{0}\right)\left(\xi_{j}-e^{-\theta j}\xi_{0}\right)\right]\\ = & \frac{1}{n^{2H}}\sum_{i,j=1}^{n}E\left[\xi_{i}\xi_{j}\right]-\frac{2}{n^{2H}}\sum_{i,j=1}^{n}e^{-\theta i}E\left[\xi_{j}\xi_{0}\right]+\frac{1}{n^{2H}}{\left(\sum_{i=1}^{n}e^{-\theta i}\right)}^{2}E{\left[\xi_{0}\right]}^{2}\\ = & \frac{r_{\xi }\left(0\right)}{n^{2H-1}}+\frac{2}{n^{2H}}\sum_{i=1}^{n-1}\sum_{j=i+1}^{n}r_{\xi }\left(j-i\right)-\frac{2}{n^{2H}}\sum_{i,j=1}^{n}r_{\xi }\left(j\right)e^{-\theta i}+\frac{r_{\xi }\left(0\right)}{n^{2H}}{\left(\sum_{i=1}^{n}e^{-\theta i}\right)}^{2} \end{array}$$

Now, in order to show the convergence (A1), we will verify that each part on the right hand side of the last equation converges to $\frac{1}{\theta^{2}}$, as *n* tendsto *∞*.

$$\begin{array}{cc} \; & \left|\frac{2}{n^{2H}}\sum_{i=1}^{n-1}\sum_{j=i+1}^{n}r_{\xi }\left(j-i\right)-\frac{1}{\theta^{2}}\right|=\left|\frac{2}{n^{2H}}\sum_{k=1}^{n-1}\left(n-k\right)r_{\xi }\left(k\right)-\frac{1}{\theta^{2}}\right|\\ \; & =\left|\left[\frac{2}{n^{2H-1}}\sum_{k=1}^{n-1}r_{\xi }\left(k\right)-\frac{2H}{\theta^{2}}\right]-\left[\frac{2}{n^{2H}}\sum_{k=1}^{n-1}kr_{\xi }\left(k\right)-\frac{2H-1}{\theta^{2}}\right]\right| \end{array}$$

According to Proposition A1, we have $r_{\xi }\left(k\right)∼k^{2H-2}$ for large *k*; then as $H>\frac{1}{2}$ and n⟶+∞, we have $\sum_{k=1}^{n}k^{2H-2}∼\frac{n^{2H-1}}{2H-1}$, which provides the following.

$$\begin{array}{c} \lim_{n\to \infty }\left|\left[\frac{2}{n^{2H-1}}\sum_{k=1}^{n-1}r_{\xi }\left(k\right)-\frac{2H}{\theta^{2}}\right]\right|⟶0. \end{array}$$

On the other hand, as n⟶+∞, we have $kr_{\xi }\left(k\right)∼k^{2H-1}$, but $\sum_{k=1}^{n}k^{2H-1}∼\frac{n^{2H}}{2H}$, as n⟶+∞. Therefore, we conclude the following

$$\begin{array}{c} \lim_{n\to \infty }\left|\left[\frac{2}{n^{2H}}\sum_{k=1}^{n-1}kr_{\xi }\left(k\right)-\frac{2H-1}{\theta^{2}}\right]\right|⟶0. \end{array}$$

Similarly, we obtain, as n→+∞, the following.

$$\begin{array}{c} \left|\frac{2}{n^{2H}}\sum_{j=1}^{n}r_{\xi }\left(j\right)\sum_{i=1}^{n}e^{-\theta i}\right|\leq Cn^{-1}⟶0 \end{array}$$

Finally, we have the following:

$$\begin{array}{c} \frac{4H\Gamma (2H)}{\theta^{2H}n^{2H}}\sum_{i,j=1}^{n}e^{-\theta (i+j)}\leq Cn^{-2H}⟶0 \end{array}$$

as n→+∞. This completes the proof of the second part. Hence, the Lemma is proved.   □
